# Behavioral Parent Training for Families With Young Deaf or Hard of Hearing Children Followed in Hearing Health Care

**DOI:** 10.1044/2022_JSLHR-22-00055

**Published:** 2022-08-19

**Authors:** Christina R. Studts, Julie A. Jacobs, Matthew L. Bush, Joneen Lowman, Philip M. Westgate, Liza M. Creel

**Affiliations:** a Department of Pediatrics, University of Colorado Anschutz Medical Campus, Aurora; b Department of Otolaryngology - Head and Neck Surgery, University of Kentucky, Lexington; c Department of Communication Sciences and Disorders, University of Kentucky, Lexington; d Department of Biostatistics, University of Kentucky, Lexington; e Department of Health Management and Systems Sciences, University of Louisville, KY

## Abstract

**Purpose:**

It is well established that individuals with a communication disability, including being deaf or hard of hearing (DHH), experience inequities in health services and outcomes. These inequities extend to DHH children's access to psychosocial evidence-based interventions (EBIs). Behavioral parent training is an EBI that can be used to improve caregiver and child outcomes. Despite being supported by decades of effectiveness research, this EBI is rarely accessed by, or studied with, caregivers of DHH children. The purpose of this article is to describe a program of stakeholder-engaged research adapting and assessing behavioral parent training with caregivers of young DHH children followed in hearing health care, aimed at reducing inequities in access to this EBI.

**Method:**

The first section briefly summarizes the literature on disruptive behavior problems in young children, with a focus on preschool-age DHH children. The evidence base for behavioral parent training is described. Next, the gaps in knowledge and practice regarding disruptive behaviors among DHH children are highlighted, and the potential integration of behavioral parent training into the standard of care for this population is proposed.

**Conclusions:**

Young DHH children who use hearing aids and/or cochlear implants experience disruptive behavior problems at rates at least as high as typically hearing children, but their access to EBIs is limited, and behavioral parent training programs tailored to this population have not been rigorously tested. Caregivers and hearing health care service providers affirm the potential benefits of behavioral parent training and were partners in adapting this EBI. This research highlights several principles and approaches essential for reducing inequities and improving the quality of life not only for DHH children and their families but also for individuals with communication disabilities more broadly: engagement of key stakeholders in research, collaboration across disciplines, and using implementation science methods and models to design for implementation, dissemination, and sustainment.

**Presentation Video:**

https://doi.org/10.23641/asha.21215900

Disruptive behavior problems are one of the most common psychosocial issues in young children, with 20% of preschool-age children exhibiting clinically significant challenging behaviors such as noncompliance, aggression, and other forms of acting out (Perou et al., 2013). The etiology of clinically significant disruptive behavior problems in early childhood (i.e., those meeting a threshold of severity for diagnosis or mental health treatment) is not well understood but is thought to include biologic and environmental contributors, including possible genetic influences, innate temperament, and exposure to environmental stressors (Loeber et al., 2009). Some disruptive behaviors are developmentally typical in young children, but when their frequency and intensity are high enough to interfere with family functioning, they are described as causing impairment and may require clinical intervention to resolve (Wakschlag et al., 2007). However, even developmentally typical disruptive behaviors can cause distress and reduce caregivers' and children's quality of life, highlighting the importance of both prevention and treatment of young children's disruptive behavior problems (Webster-Stratton, 1998).

Child disruptive behaviors are associated with high levels of caregiver stress, low levels of perceived parenting competence and satisfaction, and lower family quality of life (see the study of Weber et al., 2019, for a review). They frequently co-occur with other (often undetected) comorbidities, including anxiety, depression, and developmental delays (Perou et al., 2013). Longitudinal studies reveal that, if not successfully addressed, emergence of clinically significant disruptive behaviors in early childhood can lead to later detriments in educational, social, economic, and health outcomes (Pelham et al., 2007; Reinke et al., 2012; Schaeffer et al., 2006).

A large body of research suggests that young deaf or hard of hearing (DHH) children who use hearing aids (HAs) and/or cochlear implants (CIs) are at least as likely as children with typical hearing to experience disruptive behavior problems, if not more so (Fiorillo et al., 2017; Hindley et al., 1994; Stevenson et al., 2015). As in children with typical hearing, some disruptive behaviors may be developmentally appropriate, whereas some may be of clinical concern. The distinction between developmentally appropriate and clinically significant disruptive behaviors is made based on their frequency, intensity, and duration in the context of a child's development. Wakschlag et al. (2014) and Charach et al. (2017) provide examples of developmentally appropriate versus clinically significant levels of noncompliance, aggression, and temper loss in preschool-age children; for example, a preschool-age child saying “no” when instructed to do something is likely developmentally appropriate, but a preschool-age child refusing to hold a caregiver's hand and running into the street may be clinically significant. Similarly, a preschool-age child who loses her temper when tired, hungry, or sick may be developmentally appropriate, but a preschool-age child with daily temper tantrums that last more than 5 min may be clinically significant (Charach et al., 2017; Wakschlag et al., 2014). In general, disruptive behaviors that are developmentally atypical, occur across settings, and cause impairment or distress to the child, caregiver, and/or others are of heightened concern (American Psychiatric Association, 2013).

Language delays and difficulties with oral communication are often cited as contributing factors to disruptive behavior problems (Barker et al., 2009; Stevenson et al., 2010), not only among DHH children but also among children with typical hearing as well (Levickis et al., 2018). Additional underlying causes of disruptive behaviors have been posited related to the specific experiences of DHH children who use HAs and/or CIs and receive speech and language interventions (Barker et al., 2009), including sensory deprivation (Horn et al., 2005), sensory overload (Hogan et al., 2011), poor caregiver–child communication (Barker et al., 2009; Lam & Kitamura, 2010), language deprivation (Black & Glickman, 2006; Humphries et al., 2016), and others. Level of hearing loss and type of auditory rehabilitation have also been linked to prevalence of disruptive behavior problems, though findings have not been consistent (Bigler et al., 2019; Theunissen et al., 2014). Furthermore, as in children with typical hearing, disruptive behavior problems in young DHH children can co-occur with other conditions, including developmental, mood, or anxiety disorders (Theunissen et al., 2015; Wong et al., 2018); however, none of these are necessary for the development of disruptive behavior problems, and many DHH children with behavioral issues have no other identified comorbidities.

## An Evidence-Based Intervention for Child Disruptive Behaviors: Behavioral Parent Training

While the causes of disruptive behaviors in early childhood are multifactorial, theory-based research demonstrates that many challenging behaviors can be reduced or prevented with the strategic use of behavioral principles. Social cognitive theory (Bandura, 1977), coercion theory (Patterson et al., 1989), and others highlight the importance of the behavioral principles of reinforcement, punishment, and extinction in whether a specific behavior is maintained, increased, or reduced. According to each of these theories, children and caregivers develop patterns of behavior over time that can be coercive, maintaining and even exacerbating negative child *and* caregiver behaviors. Caregivers' reactions to child behaviors can shape future behaviors: Caregivers can unintentionally reinforce negative behaviors (e.g., the classic example of “giving in” to stop a tantrum), can extinguish negative *or* positive child behaviors by ignoring them, or can increase prosocial and desirable child behaviors through strategic positive and negative reinforcement. Similarly, children's responses to caregivers' behaviors can reinforce parenting choices (e.g., “giving in” stops the tantrum and escalating to yelling elicits compliance), resulting in repeated use of ineffective and potentially detrimental parenting strategies. These caregiver–child interactions comprise a negative, self-perpetuating cycle that can be difficult to break (Dishion & Patterson, 2006; Patterson & Yorger, 2002).

Behavioral parent training is an evidence-based intervention (EBI) to prevent or treat disruptive behavior problems in children and adolescents (Kaminski & Claussen, 2017). By interrupting coercive cycles of caregiver–child interactions, this intervention can change that trajectory, improving a range of child and family outcomes. First developed in the 1960s to help mothers of children with developmental disabilities address their children's behavioral challenges (Kaehler et al., 2016), behavioral parent training programs have demonstrated efficacy and effectiveness over decades of study with caregivers and children (Kaehler et al., 2016; Lundahl et al., 2006). Outcomes include reduced child disruptive behaviors, reduced stress and depressive symptoms in caregivers, and increased satisfaction and perceived competence among caregivers (Furlong et al., 2013). Positive outcomes in parenting and child behaviors have been documented well over 10 years after intervention delivery (Kaminski & Claussen, 2017), and this EBI has been established as cost-effective in averting future negative outcomes requiring higher intensity interventions (Dretzke et al., 2005; Furlong et al., 2013).

Behavioral parent training is typically delivered by a trained mental health professional to individual caregivers or to groups of caregivers. Depending on the program, the intervention typically includes up to 14 sessions, each lasting 1–2 hr. The location of program delivery is also variable, with sessions offered by organizations, agencies, or individual providers in mental health centers, schools, private offices, homes, and remotely. Numerous versions of behavioral parent training programs exist (e.g., Parent–Child Interaction Therapy [Lieneman et al., 2017], the Incredible Years [Webster-Stratton, Rinaldi, & Jamila, 2011], Triple-P [Li et al., 2021], and the Family Check-Up [FCU; Dishion et al., 2014]), and they share in common a focus on improving caregiver–child interactions, training caregivers to use positive behavioral supports (e.g., positive reinforcement for desired behaviors) and effective limit-setting strategies (e.g., consistent rules and consequences), and using training methods (e.g., modeling, role-play, video feedback, and homework) to maximize caregivers' mastery of skills (Chorpita & Daleiden, 2009).

While demonstrated effective and recommended for widespread use, behavioral parent training is underdelivered and difficult to access in the United States (Comer & Barlow, 2014; Dodge, 2018). Fewer than 20% of children and caregivers who could benefit from this EBI are estimated to receive it (Kazdin & Blase, 2011; Perou et al., 2013), and at least 25% drop out of behavioral parent training programs that they begin (Chacko et al., 2016). Barriers to its reach are multilevel. At the family level, perceived stigma, financial constraints (e.g., lack of transportation to service locations, challenges affording out-of-pocket payments for this service), relative priority (e.g., other social needs in the family taking precedence), and lack of knowledge about child behavior are common barriers to seeking intervention (Koerting et al., 2013). At the organizational level, adoption of EBIs is often lacking due to competing demands and limited resources (Kohl et al., 2009), and many behavioral providers are not trained in this approach (Kazdin & Blase, 2011). At the societal level, systems serving families and children are chronically underfunded and overburdened (Fagan et al., 2019), and access to EBIs is limited for families in poverty, families who are members of racial or ethnic minorities, and families in rural communities (Baumann et al., 2015; Kazdin & Blase, 2011). This constellation of structural and cultural barriers reduces equitable access to psychosocial interventions, limiting the potential public health impact of behavioral parent training and other EBIs.

## Behavioral Parent Training and Deaf and Hard of Hearing Children

Given this limited access to behavioral parent training in the general population, it is perhaps unsurprising that DHH children and their families are especially unlikely to receive this EBI. Lack of access to mental health services in general is a known disparity for DHH individuals, and DHH children are no exception (Vernon & Leigh, 2007). Providers of mental health services to DHH individuals should be linguistically and culturally competent (Vernon & Leigh, 2007), and in a country with widespread shortages of *any* mental health providers (U.S. Department of Health & Human Services, 2022), these training and experiential backgrounds are rare. Mental health services were highlighted nearly 4 decades ago as the most requested but least available service for DHH individuals (Wilson & Schild, 2014), and progress in this area has been inadequate.

Early in our program of research with DHH children and followed in hearing health care, we assessed levels of disruptive behaviors among preschool-age children who were DHH and used either HAs (*n* = 29) or CIs (*n* = 21), compared to peers with typical hearing (*n* = 39; Fiorillo et al., 2017). Using a structured clinical diagnostic interview (Fisher & Lucas, 2006) and controlling for oral language development (Fenson et al., 2007), we found that 4 times as many caregivers of children with HA and CI than caregivers of children with typical hearing reported being concerned about their child's emotional and behavioral functioning (41% and 38% vs. 10%), over twice as many endorsed the presence of behavioral symptoms that met the diagnostic criteria for oppositional defiant disorder (48% and 48% vs. 23%), and 3 times as many reported impairment associated with these symptoms (43% and 48% vs. 15%). Despite these clear indications of behavioral concerns, however, no caregivers of children who were DHH reported receiving mental health or behavioral services for their child, compared to 8% of caregivers of children with typical hearing. In the general population, caregivers of children with disruptive behavior problems are much more likely to seek mental health services (Johnston & Burke, 2020), compared to caregivers of children with other emotional and behavioral issues, but these services do not seem to reach caregivers of DHH children.

This gap appears not only in practice but also in the research underlying services to DHH children and their families. Despite its origins with mothers of children with disabilities, research on the efficacy and effectiveness of behavioral parent training with caregivers of children with disabilities is quite limited (Totsika et al., 2017), and when that scope is narrowed to caregivers of DHH children, research is almost nonexistent. A few studies address associations between parenting and child outcomes in this population (e.g., Barker et al., 2009; Quittner et al., 2010) and several case studies of behavioral parent training with caregivers of DHH children have been published (e.g., Shinn, 2013), but there are no rigorous experimental trials testing the effects of behavioral parent training with caregivers of DHH children followed in hearing health care. As articulated in the 2008 Position Statement on Mental Health Services for Deaf Children by the National Association of the Deaf, “evidence-based practices are largely untested for low incidence populations, such as deaf [including hard of hearing, late deafened, and deaf-blind] children. Because of the mental health system's failure to address the unique needs of deaf children, they are subject to increased risks and barriers to their mental health.” It is long past time to address this inequity in care.

## Could Behavioral Parent Training Be Incorporated Into the Standard of Care for Deaf and Hard of Hearing Children and Their Families?

Disruptive behaviors in early childhood are common, whether developmentally typical or clinically significant. Delivery of behavioral parent training to caregivers of young children can be preventive (i.e., addressing developmentally typical disruptive behaviors to prevent them from worsening) or for treatment of clinically significant disruptive behavior problems, and positive results are reported for both approaches (Kaminski & Claussen, 2017). Given the high prevalence of disruptive behavior problems reported by caregivers of DHH children, as well as the usual stressors of young children's challenging behaviors that may be developmentally expected, behavioral parent training for either preventive or treatment purposes could improve child behavior, parenting behaviors, and related outcomes in this population.

**Figure 1.**
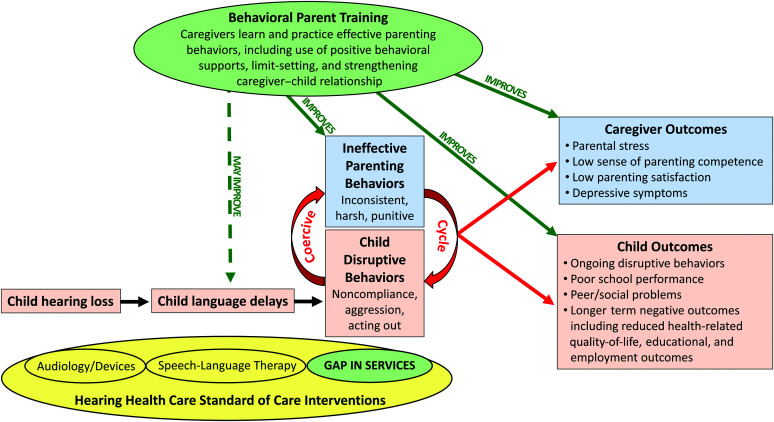
Behavioral parent training as an intervention to fill a gap in hearing health care services for young deaf and hard of hearing children and their caregivers. This figure depicts a likely sequence from (a) child hearing loss to (b) delays in child language development, leading to (c) disruptive behavior problems in some young DHH children. The interventions comprising the standard of care for most young DHH children followed in hearing health care include audiology services and devices (i.e., hearing aids, cochlear implants, and bone-conduction devices) and speech-language therapy. There is a gap in services targeting the disruptive behavior problems reported in this population. When child disruptive behaviors are responded to with ineffective parenting behaviors, a coercive cycle develops that is challenging to break and that leads to negative child and caregiver outcomes. Behavioral parent training is proposed as an intervention to fill this identified gap in services. It has been shown to improve parenting behaviors, child outcomes, and caregiver outcomes in families with children with typical hearing, and may improve child language outcomes.

The standard of care for young DHH children with auditory rehabilitation usually includes HAs, CIs, or other devices; speech-language therapy; and, for some children, additional early intervention services (e.g., physical therapy and occupational therapy). None of these interventions intentionally target the caregiver–child interaction cycle that can maintain or exacerbate children's behavioral problems. To complement the array of services caregivers are able to access for young DHH children, behavioral parent training could provide caregivers with effective strategies to improve caregiver–child interactions, reduce and prevent disruptive child behaviors, improve parenting behaviors, and improve caregivers' sense of competence and satisfaction with the parenting role (see Figure 1). Integration of behavioral parent training into the standard care for DHH children and their families could have a significant impact on their long-term outcomes.

Beyond the expected benefits of behavioral parent training, recent studies have highlighted potential spillover effects of this EBI, several of which could be especially desirable for DHH children and followed in hearing health care. In a study of the effects of Parent Child Interaction Therapy delivered to caregivers of young children with disruptive behavioral problems and developmental delay, improvements in parenting skills led to significant increases in children's productive vocabulary (Garcia et al., 2015); similar improvements in infant language production were reported in a trial of this behavioral parenting intervention with infants (Bagner et al., 2016). Multiple studies have shown that behavioral parent training yields improvements not only in disruptive behavior problems but also in children's symptoms of anxiety and depression, as well as caregivers' depressive symptoms and perceived stress (Carpenter et al., 2014; Gonzalez & Jones, 2016; Webster-Stratton & Herman, 2008)—all highly relevant to children with DHH and their families.

Finally, young children's resistance to wearing HAs, CIs, or other devices is commonly described by caregivers, who sometimes struggle to meet audiologists' recommendations for wear time (Moeller et al., 2009; Walker et al., 2013, 2015) and report inadequate supports and guidance from hearing health care providers in addressing this issue (Muñoz et al., 2016). Given the importance of high-quality linguistic input to children's oral language development, adherence to wearing devices is crucial in this population (McCreery et al., 2015; Tomblin et al., 2014; Wang et al., 2011), but evidence-based approaches supporting caregivers in ensuring adherence are lacking (Ambrose et al., 2020). Many providers reportedly lack skills and confidence in addressing this problem (Harrison et al., 2016). Behavioral parent training is specifically designed to empower caregivers to identify specific child behaviors to target with effective reinforcement strategies, making it a potentially powerful tool for increasing device use and, thus, aural/oral linguistic input and language development (Tomblin et al., 2014; Wang et al., 2011).

## An Implementation Science Approach to Research on Behavioral Parent Training for Deaf and Hard of Hearing Children and Their Families

Should behavioral parent training be incorporated into the standard of care for young DHH children who use HAs and/or CIs? Answering this question begins with three important tasks: (a) evaluating perspectives and buy-in about the perceived need for parenting support among caregivers of young DHH children and other key stakeholders; (b) assessing whether and how this EBI should be adapted to meet the needs of DHH children and their families; and (c) testing the effects of behavioral parent training with the target population in real-world settings. In this article, we describe a program of research illustrating the first two of these tasks, and we introduce ongoing work addressing the third.

The field of implementation science offers principles, frameworks, and methods well aligned with these tasks. Perhaps most importantly, the practice of stakeholder engagement throughout all phases of intervention research, including needs assessment, planning, implementing, and evaluating interventions, ensures that those with vested interests in and influence upon the potential delivery of EBIs are collaborators in their development and testing (Forsythe et al., 2016). In the case of behavioral parent training for DHH children and their families, key stakeholders may include caregivers, DHH adults, and a range of service providers, including audiologists, speech-language pathologists, Deaf educators, early interventionists, and others. Engaging multilevel stakeholders, from the intended recipients of an intervention to experts in related fields and policy-makers, increases not only the breadth of perspectives incorporated into each phase of intervention research but also transparency and accountability in the participatory planning, implementing, and evaluating of interventions (Boaz et al., 2018), particularly with marginalized groups and communities (Baumann & Cabassa, 2020).

The implementation science principle of *designing for dissemination, implementation, and sustainment* should also guide efforts to adapt, test, and deliver interventions such as behavioral parent training for caregivers of DHH children (Brownson et al., 2013; Chambers, 2020; Kwan et al., 2022). Despite good intentions, many interventions are developed and tested in ways that limit their potential for use outside of research settings. In reality, EBIs that have only been developed and tested in academic, controlled settings are unlikely to translate easily to agencies, organizations, and communities with variable characteristics, priorities, and resources (Feldstein & Glasgow, 2008; Glasgow et al., 2019). Those developed without attention to the preferences and perspectives of intended recipients and deliverers are unlikely to be accessed or adopted (Proctor et al., 2011), and those requiring expertise, supports, or infrastructures beyond what is available in the context of usual care are unlikely to be implemented or sustained (Fagan et al., 2019; Feldstein & Glasgow, 2008; Shelton et al., 2020). By ensuring that a behavioral parent training intervention adapted for caregivers of DHH children is acceptable, feasible, and sustainable, an implementation science approach increases the potential public health impact of this EBI for a new population and service setting.

Systematic models for adapting existing EBIs are also promoted in implementation science (Escoffery et al., 2019), seeking to ensure that modifications made to effective interventions increase their fit with new populations and contexts while preserving components thought to be essential for effectiveness. Implementation science offers multiple models for assessing the need for adaptation, guiding adaptation decisions, and tracking both planned and unplanned adaptations to EBIs (Stirman et al., 2019). Use of these models promotes transparency and contributes to our understanding of “what works, for whom, and under what conditions,” reinforcing the facts that (a) context matters and (b) a one-size-fits-all approach may not lead to desired outcomes (Chambers & Norton, 2016).

Finally, implementation science emphasizes the need to assess different outcomes than the recipient-level effects traditionally measured in intervention research. Implementation frameworks, such as the Implementation Outcomes Framework (Proctor et al., 2011) and the Reach, Effectiveness, Adoption, Implementation, and Maintenance (RE-AIM) framework (Glasgow et al., 1999, 2019), capture important effects of implementing EBIs, including their acceptability, feasibility, and appropriateness from stakeholders' perspectives (Proctor et al., 2011; Weiner et al., 2017); reach (i.e., what proportion of eligible people receive it); adoption by interventionists and organizations; fidelity, adaptations, and costs of implementation; and maintenance of intervention delivery and effects. Intertwined within each of these outcomes is the issue of representativeness (Shelton et al., 2020): Is there equity in who does and does not receive, deliver, benefit from, and sustain an EBI and its effects? Hybrid effectiveness–implementation trials are an implementation science innovation that allow the simultaneous consideration of effectiveness outcomes and implementation outcomes at each stage of intervention and implementation research (Curran et al., 2012; Landes et al., 2019). In determining the potential impact of behavioral parent training for caregivers of young DHH children, caregiver- and child-level outcomes must be investigated, but so too must outcomes including feasibility, acceptability, fidelity, and costs of the intervention (Proctor et al., 2011), and if established as effective, future research should address the implementation strategies needed to ensure broad and representative reach, adoption, implementation, and maintenance (Glasgow et al., 2019) of this EBI, especially given what we know of limited access to other mental health interventions for individuals who are DHH.

## (a) Evaluating Perspectives and Buy-in About the Perceived Need for Parenting Support Among Caregivers of Young DHH Children and Other Key Stakeholders

Following our initial study of the prevalence of behavioral issues among young DHH children and followed in hearing health care (Fiorillo et al., 2017), we conducted key informant interviews with 39 caregivers of young DHH children. Semistructured interview guides explored caregiver perceptions of their parenting experiences, including confidence and satisfaction; perceptions of their DHH child's emotional and behavioral functioning; and the need for and/or availability of parenting resources and supports through community, educational, and hearing health care organizations. While some caregivers described adequate support systems, easy access to supportive professionals, and few behavioral concerns regarding their children, many expressed feeling isolated, lacking resources and knowledge about child development and behaviors, and questioning whether they were effectively meeting their children's emotional and behavioral needs. When asked about available resources to provide needed parenting information and support for caregivers of DHH children, no existing resources were identified.

Based on the results of this formative work, we established a Hearing and Behavior Community Advisory Board (CAB) in 2017, inviting a wide range of potential members and asking invitees to invite others who may be interested. Invitations to the initial CAB meeting were extended to approximately 20 community members, including caregivers of DHH children, Deaf adults, audiologists, speech-language pathologists, DHH mental health providers, Deaf educators, early interventionists, and administrators of state-level offices serving DHH children. Through the first 2 years of this program of research, approximately 12 core members of the CAB reviewed results of our formative work, made recommendations regarding steps forward, and informed decisions regarding research design and procedures.

## (b) Assessing Whether and How This EBI Should be Adapted to Meet the Needs of Deaf and Hard of Hearing Children and Their Families

Prior to making decisions about the need for adaptations to behavioral parent training to meet the needs and preferences of caregivers of DHH children, we conducted a small pilot study of an “off-the-shelf” behavioral parent training intervention: the FCU (Dishion et al., 2008, 2014; Shaw et al., 2006). The FCU is unique among behavioral parent training interventions in its flexibility and tailoring to individual families' needs. While most behavioral parent training interventions involve 12–14 sessions addressing a linear curriculum of parenting topics, the FCU begins with two to three sessions of structured assessment and feedback, followed by joint selection of prioritized skills and sessions by the interventionist and caregivers. This flexibility and potential for brevity is desirable for an intervention that could be used preventively or clinically and prompted our initial selection of the FCU versus other behavioral parent training interventions.

For the pilot trial, we recruited 12 adult caregivers (age 18 years or older) of DHH children (ages 2–5 years) who used HAs and/or CIs from local and regional hearing health care clinics. All caregivers were biological parents of a DHH child and were hearing; several used American Sign Language (ASL) and oral communication with their children. See Table 1 for additional child and caregiver participant characteristics. The goal of the pilot study was to determine the feasibility of study procedures for a subsequent randomized trial of the FCU, including assessment of caregiver satisfaction with the intervention. Caregivers were stratified by child's type of hearing device and randomized in a 1:1 ratio to intervention (an abbreviated three-session FCU) or attention–control (a family healthy eating intervention for caregivers). The attention–control condition was included to ensure that time spent with an interventionist was comparable between the two groups, and the healthy eating intervention was selected as an attention–control condition that would not include or address content about child behavior or parenting strategies. Caregivers in each group completed three weekly individual meetings with an interventionist (trained doctoral students in clinical psychology) and completed post-intervention measures.

**Table 1.** Pilot trial participants (*N* = 12 parent–child dyads).

Characteristic	Intervention (FCU)	Control (healthy eating)
Child amplification device		
Hearing aids	3	3
Cochlear implants	3	3
Child mean age (years)	4 years 0 months	3 years 8 months
Child race		
White	4	4
Black	1	1
More than one race	1	1
Child ethnicity		
Non-Hispanic	6	6
Child sex		
Male	3	1
Female	3	5
Parent mean age (years)	35	32
Parent sex		
Male	1	2
Female	5	4
Parent education		
Some college	4	1
College degree	1	1
More than a college degree	0	3
Median annual household income range	$70,000–$80,000	$30,000–$40,000

*Note.* FCU = Family Check-Up.

Pilot administration of measures included multiple caregiver-completed instruments, including a sociodemographic background questionnaire, the Child Behavior Checklist/1–5 (Achenbach & Ruffle, 2000), the Parenting Sense of Competence Scale (Gilmore & Cuskelly, 2009), the Impact on Family Scale (Stein & Jessop, 2003), the Parenting Stress Index-4 (Abidin, 1995), the MacArthur–Bates Communicative Language Development Inventory (Fenson et al., 2007), the Parenting Young Children Scale (McEachern et al., 2012), and the European Parent Satisfaction with Early Intervention Services (EPASSEI) measure (Lanners & Mombaerts, 2000).

Feasibility of the study procedures was supported by successful recruitment of the planned sample size, completion of all baseline measures, and retention of five out of six FCU participants through post-intervention assessment. Results of caregiver responses to the EPASSEI revealed that five of six FCU participants provided a mean satisfaction rating of 4 or higher (on a 5-point Likert scale) for the scale's 20 items. Open-ended feedback provided by caregivers was similarly positive, voicing appreciation of learning new skills and receiving insights and direction from the FCU interventionists; however, brief follow-up interviews with several caregivers from the pilot study indicated that the lack of representation of DHH children and the lack of inclusion of common behavioral scenarios faced by their caregivers could limit the value of the intervention.

This feedback suggested that adaptations to the FCU could increase its relevance and acceptability to caregivers of DHH children. We followed the eight-phase ADAPT-ITT model (Wingood & DiClemente, 2008) to identify and incorporate systematic adaptations to the original intervention. Stages of ADAPT-ITT include assessment (of members of the target population and key stakeholders), decision (on an intervention and needed adaptations), administration (obtaining feedback on proposed adaptations), production (incorporating stakeholder feedback into an initial adapted draft intervention), topical experts (consultation with experts in the original intervention to ensure that proposed adaptations do not compromise presumptive core components), integration (finalizing adaptations), training (training intervention and research staff on the adapted intervention), and testing (conducting a trial of the adapted intervention; Wingood & DiClemente, 2008).

In the assessment phase, we conducted additional key informant interviews with 20 caregivers of DHH children ages 3–7 years. A semistructured interview guided the participant to describe preferences and share recommendations for the delivery and content of a behavioral parent training intervention designed to meet the needs of this population. Interviews were recorded and transcribed verbatim and then coded line-by-line by two qualitatively trained independent coders who discussed discrepancies and reached consensus through discussion. Regarding intervention delivery, overall caregiver preferences were for interventionists with experience with DHH children, individual rather than group sessions, in-home session delivery, and no more than six sessions. Specific content recommended to be added to a behavioral parent training intervention targeting caregivers of young DHH children included emphasizing communication strategies and methods; incorporating behavioral scenarios commonly experienced in families of DHH children; providing emotional and informational support regarding hearing loss; including specific information about child development and DHH children; and addressing advocacy needs and strategies.

Next, we conducted four focus groups with 12 service providers to DHH children and families, with separate groups for audiologists, speech-language pathologists, and Deaf educators. Focus group guides included open-ended questions eliciting perspectives on the prevalence of behavioral problems among preschool-age DHH children served, specific skills or strategies that could be particularly beneficial for caregivers of DHH children, and recommendations regarding delivery of a behavioral parent training intervention. Focus group discussions were recorded and transcribed verbatim. Service providers estimated that at least half of their DHH child clients exhibited behavioral problems at home and in other settings, confirming both the potential need for interventions targeting caregivers' responses to negative child behaviors and the lack of resources and services addressing this need. Specific topics emphasized by services providers during focus group discussions included caregivers' challenges in addressing child refusal to wear devices, the negative impact of caregiver and child frustration with communication barriers, caregivers' struggles with consistency and follow-through when addressing child behaviors, the potential benefits of home-based intervention delivery, and the need for caregiver education on advocacy.

Qualitative findings from caregiver interviews and service provider focus groups were presented to the CAB for additional feedback and brainstorming. An essential contribution of the CAB was to review, recommend, and assist with making adaptations to the existing FCU delivery protocol and to the content of parenting sessions. Final adaptations included (a) partnering with the state chapter of Hands & Voices to select and train “parent coaches” who were themselves caregivers of DHH children to serve as interventionists; (b) revising role-play exercises and example scenarios in the FCU curriculum to focus on preschool-age DHH children and frequently mentioned experiences (e.g., refusing to wear HAs or CIs; caregiver being unsure if child has heard an instruction; child being fatigued or irritable after a full day of wearing devices at child care or preschool; caregiver conflicts with siblings over discrepant expectations for DHH child); and (c) compiling existing informational handouts, guides, websites, and other resources for parent coaches and caregivers on (1) oral communication strategies, ASL training, and other communication resources; (2) developmental milestones and language development in DHH children; and (3) advocacy and self-advocacy strategies for DHH children. The additional resources compiled for parent coaches and caregivers were designed to be integrated into parenting sessions as appropriate but could also be provided to caregivers by parent coaches outside of sessions if desired.

## (c) Testing the Effects of Behavioral Parent Training With Caregivers of Young Deaf and Hard of Hearing Children in Real-World Settings

The current stage of our program of research is a hybrid effectiveness–implementation trial of the adapted FCU (called the FCU-DHH) with the target population (http://clinicaltrials.gov NCT03916146; Studts et al., 2022). The FCU-DHH incorporates the adaptations described previously, in addition to the assessment-focused, strengths-based, and tailored content and methods of the original FCU behavioral parent training intervention. The intervention is delivered by “parent coaches,” who are themselves caregivers of DHH children, employed in the Hands & Voices Guide by Your Side program (a parent-to-parent support program for caregivers of DHH children; see https://www.handsandvoices.org/gbys/index.htm) and trained and supervised in the FCU-DHH. This ongoing trial, funded by the National Institute of Deafness and Other Communication Disorders, uses mixed methods and a prospective randomized controlled trial design to (a) test the effects of the FCU-DHH on parenting behavior and child behavior; (b) assess secondary effects of the intervention on child adherence to wearing devices and on a range of child language outcomes; (c) explore implementation outcomes, including acceptability, adoption, feasibility, fidelity, and costs of the FCU-DHH; and (d) explore contextual factors, including caregiver and parent coach perceptions of the intervention; parent coach characteristics and skills; compatibility of the FCU-DHH with Hands & Voices priorities and supports; and other factors that may serve as barriers or facilitators to future dissemination and implementation of this intervention, if found to be effective. Data will be collected from caregivers, children, parent coaches, and other stakeholders over a 3-year period.

The Hearing and Behavior CAB continues to provide valuable insights as the trial progresses, assists with sharing recruitment information with hearing health care practices and other organizations across the state, and will contribute to dissemination of results as they become available. To our knowledge, this is the first rigorous trial of a behavioral parent training intervention for DHH children and their caregivers, and the first hybrid effectiveness–implementation trial investigating the effects and potential for implementation of an EBI with this population (Studts et al., 2022).

## Summary and Future Directions

Inequitable access to evidence-based care is an persistent reality in the United States and one that must be addressed with research, practice, and policy. Marginalized populations experience disparities in services and outcomes and include (but are not limited to) those in poverty; those who are minoritized due to race, ethnicity, sexual orientation, or gender identity; those with disabilities; and those with intersecting identities among these groups. Increasing equitable access to evidence-based care and subsequent health, mental health, and quality-of-life outcomes should be a prominent objective of researchers, practitioners, and policy-makers. The program of research described here addresses just one example of this societal problem: the lack of research on and delivery of behavioral parent training for caregivers of young DHH children. Numerous studies report elevated rates of behavioral problems among DHH children who use HAs and/or CIs (Fiorillo et al., 2017; Hindley et al., 1994; Stevenson et al., 2015), but even if disruptive behaviors occurred in this population at the identical rate observed in children with typical hearing, the continued lack of access to EBIs for prevention and treatment of child mental health problems is a failure of our child-serving systems and the policies that impact them (Dodge, 2018; Kazdin & Blase, 2011; Stewart et al., 2016).

Compared to caregivers of young children with typical hearing, caregivers of young DHH children followed in hearing health care are more likely to report disruptive behavior problems; impairment in caregiver, child, or family functioning associated with those behaviors; and not having received mental health or behavioral interventions for their child. There is an EBI that considered the standard of care for disruptive behavior problems in children: behavioral parent training, an intervention that has existed for over 50 years (Kaehler et al., 2016). Despite caregivers' experiences and concerns (Muñoz et al., 2021; Quittner et al., 2010), the prevention and treatment of disruptive behavior problems in DHH children has been a low priority, as evidenced by a dearth of rigorous intervention research and a lack of culturally and linguistically appropriate services. In our stakeholder-engaged research, caregivers of DHH children and hearing health care (and related) service providers affirm the potential benefits of behavioral parent training and have been partners in adapting this EBI to meet their unique needs.

The field of implementation science offers principles and approaches that can guide efforts to reduce inequities in care and improve outcomes not only for this population but also for individuals with other communication disorders and other disabilities more broadly. It is important to note that within the population of DHH children and their families, additional inequities exist. Disparities in access to and experiences with health care due to racism, sexism, and sexual and gender identities, for example, compound those already experienced by many individuals with hearing loss, other communication disorders, and other disabilities. The importance of recognizing the social and structural barriers to care for those with historically marginalized intersecting social identities cannot be overstated. Recent developments in implementation science and health equity research have emphasized the need to simultaneously consider multiple sources of inequities and strategies to address them at every stage of intervention development, testing, adaptation, and implementation.

The public health impact of any EBI is severely limited when it is not delivered with broad reach; is not provided equally to all who could benefit from it; is not adopted by the providers and settings where the individuals who need it access their care; is not delivered with high quality in ways consistent with the needs, preferences, and resources of recipients and settings; and is not planned for or sustained with supportive infrastructures and policies. In the case of behavioral parent training as a preventive or clinical intervention for DHH and their families, all of these systemic shortcomings exist. Implementation science frameworks and methods may inform research, practice, and policies to overcome previous failures and improve the care available to this population.

Stakeholder engagement at all stages of intervention research and implementation is a key tenet of implementation science (Goodman & Thompson, 2017). By ensuring that members of the target population, individuals in direct service and administrative roles, and decision-makers at multiple levels are involved in the development, adaptation, and implementation of interventions, their feasibility, acceptability, and potential for implementation outside of the research context are increased. Partnering with stakeholders throughout the process of developing, testing, adapting, and implementing evidence-based programs is essential in ensuring that we design for dissemination, implementation, sustainability, and equity (Kwan et al., 2022; Shelton et al., 2021). In our ongoing program of research, we have engaged caregivers of DHH children; Deaf adults; direct service services providers from audiology, speech-language pathology, mental health, education, and early intervention; and administrators in state offices serving DHH children in the selection, adaptation, and evaluation plans for the FCU-DHH behavioral parent training intervention. The shared commitment of our research team and CAB to improve the care available to DHH children and families is a strength of this research, illustrating the value of intentional integration of stakeholder input across phases of research—with the goal of impacting future practice and policy.

Similarly, transdisciplinary collaboration in intervention and implementation research is necessary for this type of work (Aarons et al., 2019). In our research on behavioral parent training for caregivers of DHH children, the training and background of our investigators is necessarily broad. Our research team brings together expertise in the academic fields of mental health, public health, social work, otolaryngology, speech-language pathology, health economics, and biostatistics, as well as a collaborator who directs our state chapter of Hands & Voices and another with leadership roles in our state Office for Children with Special Health Care Needs and Early Hearing Detection and Intervention (EHDI) program. A limitation of our current team is the lack of representation of researchers in disability disparities and Deaf studies, and we plan to address this limitation as our work moves forward.

As opposed to siloed, discipline-specific, solo investigator research of the past, research to impact practice and policy requires a team science approach crossing disciplinary and professional boundaries, integrating and valuing diversity of expertise and experiences. Other recent studies of services for DHH children and children with other disabilities have suggested additional opportunities to coalesce strategies and findings toward additional improvements to the care accessed by this population. Research on caregiver training and parent-to-parent support has targeted communication empowerment of DHH children (Nicastri et al., 2021), vocabulary enhancement (Lund, 2018), and navigation of options following diagnosis (Mehta et al., 2020), and home visiting and coaching models provide both guidance and opportunities to combine services targeting families' individual strengths and needs (Nicholson et al., 2016; Noll et al., 2021). Transdisciplinary collaborations, while sometimes challenging, may lead to leaps in understanding rather than small incremental steps.

Finally, our use of a hybrid effectiveness–implementation trial to test caregiver- and child-level effectiveness outcomes of the FCU-DHH while exploring implementation outcomes and contextual influences on its potential future adoption, implementation, and sustainment illustrates the potential of these trials to speed the translation of research to practice (Curran et al., 2012; Landes et al., 2019). Hybrid trials value both effectiveness and implementation outcomes and highlight the importance of asking practical and pointed questions early on and throughout each stage of research. In the formative work building toward our hybrid trial and during the course of the trial itself, we continue to check our assumptions and ask crucial questions about the acceptability, appropriateness, and feasibility of the FCU-DHH: Is this intervention a priority for the target population and for stakeholders? Can it be designed and delivered in ways that meet caregiver preferences and still yield expected outcomes? Do adaptations increase the fit of the intervention with those who receive it and those who deliver it? Are the costs of delivery reasonable and could this intervention be delivered and sustained outside of the research context? Will it reach and be accessible to those who need it most? By attending to these issues across each phase of research, we maintain a focus on designing for dissemination, implementation, sustainability, and equity (Kwan et al., 2022).

Through adapting and testing a behavioral parent training intervention for parents of young DHH children, this program of research aims to reduce inequities in access to needed care and related child and family outcomes. If the FCU-DHH is found to improve DHH child and caregiver outcomes as hypothesized, future directions include identifying and testing of implementation strategies to scale up delivery of the intervention outside of this study; investigating the need for further adaptations; assessing dissemination approaches through hearing health care practices; exploring the influence of varying contexts on delivery of the intervention; and posing additional research questions relevant to the reach, effectiveness, adoption, implementation, and maintenance of this intervention to increase its impact with young DHH children and their families. These recent, current, and future studies highlight several principles and approaches essential for reducing inequities not only among DHH children and their families but also among individuals with communication disability more broadly: engagement of key stakeholders in research, collaboration across disciplines, and using implementation science methods and models to promote broad and representative reach, adoption, implementation, and sustainment of evidence-based care. We invite others to integrate implementation science approaches into planning, implementing, and evaluating the delivery of evidence-based care to promote health equity among individuals with communication disabilities and their families.

## Supplementary Material

10.1044/2022_JSLHR-22-00055SMS1 Presentation Video Presentation Video
